# Effects of preservative fluid associated possible donor-derived carbapenem-resistant *Klebsiella Pneumoniae* infection on kidney transplantation recipients

**DOI:** 10.1186/s12882-022-02733-7

**Published:** 2022-03-14

**Authors:** Fei Zhang, Jinbiao Zhong, Handong Ding, Guiyi Liao

**Affiliations:** 1 Department of Urology, the First Affiliated Hospital of Anhui Medical University, Hefei, Anhui Province China; 2 Institute of Urology, the First Affiliated Hospital of Anhui Medical University, Hefei, Anhui Province China; 3 Anhui Province Key Laboratory of Genitourinary Diseases, Anhui Medical University, Hefei, Anhui Province China

**Keywords:** Donor-derived infection, Carbapenem-resistant *Klebsiella pneumoniae*, Preservation fluid, Possible donor-derived carbapenem-resistant *Klebsiella pneumoniae*, Kidney transplantation

## Abstract

**Background:**

Infections remain a major cause of morbidity and mortality in kidney transplant (KT) recipients. This study aimed to investigate the preservation fluid (PF) samples from deceased donors and report the impacts of possible donor-derived carbapenem-resistant *Klebsiella pneumoniae* (pdd-CRKP) infections on KT recipients.

**Methods:**

A retrospective study was performed that included all recipients who received kidney transplantation from deceased donors in our hospital between December 2018 and December 2020. A total of 212 patients received kidney transplantation from deceased donors, a total of 206 PF samples were collected, and 20 recipients had a CRKP-positive culture. Both donors and recipients with CRKP-positive PF cultures were divided into two groups, and continuous variables between the two groups were compared using independent-sample t tests and Mann-Whitney tests. Categorical variables were compared using the chi-square test or Fisher’s exact test. The significance level of *p* values was set at 0.05.

**Results:**

A total of 337 recipients underwent kidney transplantation, including 212 recipients of organs from deceased donors and 110 corresponding deceased donors. A total of 206 PF samples were collected, and 20 recipients had CRKP-positive PF cultures. The donors’ length of ICU stay was a potential risk factor for CRKP positivity in the PF culture (*P* < 0.05). Fifteen recipients were infected with pdd-CRKP, and the incidence of pdd-CRKP infection was 7.3% (15/206). The use of antibiotics, including ceftazidime-avibactam (CAZ-AVI), was a potential protective factor against death and graft loss in recipients with a CRKP-positive PF culture (*P* < 0.05).

**Conclusions:**

This study shows that the incidence of pdd-CRKP is high in our centre, recipients with pdd-CRKP infection can still achieve a good prognosis with the use of antimicrobial agents including CAZ-AVI.

**Supplementary Information:**

The online version contains supplementary material available at 10.1186/s12882-022-02733-7.

## Introduction

Kidney transplantation is the most effective therapy for end-stage kidney diseases [1]. However, approximately 60% of the deaths and graft losses in the first year after transplantation are caused by infection and perioperative complications [2, 3]. Infection has become a major cause of morbidity and mortality after solid organ transplantation (SOT). Bacterial infection is the most common type of infection after transplantation, with most infections occurring in the first three months after transplantation [3, 4]. An accumulation of data over the past few years confirm an increasing trend of gram-negative bacterial infections and the emergence of multidrug-resistant (MDR) bacterial pathogens [5]. An Italian study on SOT recipients showed that 16% of organ recipients were infected by gram-negative microorganisms, most of whom were infected with carbapenem-resistant *Klebsiella pneumoniae* (CRKP) [6].

In recent years, with the increase in the number of kidney transplant (KT) recipients, the gap between the number of available kidneys and the number of potential recipients has widened. The increased demand has led to increased utilisation of organs from marginal donors, including infected marginal donors [7]. Donor-derived infections (DDIs) have also become one of the major problems after SOT. Some studies reported that recipients with this type of infection could achieve favourable outcomes with appropriate antimicrobial therapy [8–11]; however, some authors suggested DDIs are associated with severe morbidity and mortality [12, 13], depending on the type of pathogenic microorganism [14]. CRKP has been reported as one of the most common pathogens in DDIs after kidney transplantation from deceased donors [15] and is characterised by high mortality, a high transmission rate and MDR [16]. Data showed that the morbidity of CRKP infection after SOT ranged from 2.5 to 35%, showing a high case fatality rate of 62.5 to 82% [17, 18].

The transmission of microorganisms through preservation fluid (PF) is a potential method for early DDI in KT recipients after KT, and routine microbial analysis of PF can help identify patients with a high risk of early post-transplantation infection [19]. Most studies have shown that coagulase-negative Staphylococcus and Enterococci are the most common contaminants in PF, most of which are low-virulence bacteria [20–22]. To date, the impact of CRKP contamination in PF on kidney recipients is not clear, and the best strategies for preventing and treating subsequent infections in kidney recipients remain unclear. Therefore, this study aimed to investigate the incidence of CRKP contamination in deceased donor PF and the impacts of possible donor-derived carbapenem-resistant *Klebsiella pneumoniae* (pdd-CRKP) infection after KT on recipients; their prevention and treatment options are also discussed.

## Materials and methods

### Study design and population

This study was a single-centre retrospective study. The case data of all KT recipients and the corresponding donors from December 2018 and December 2020 were reviewed via electronic medical records in the Kidney Transplantation Center of the First Affiliated Hospital of Anhui Medical University. Six recipients who did not have PF cultures were excluded from the study. None of these kidneys were obtained from prisoners. In this study, all kidneys were donated voluntarily after the death of the donors, and were obtained by an organ procurement organisation established by the hospital. The study was approved by our Institutional Ethics Review Committee and conducted in accordance with the Declaration of Helsinki guidelines.

Some variables were collected from the recipients’ medical records, including age, sex, body mass index, aetiology of kidney failure, delayed graft function, type of induction therapies, type of dialysis, duration of dialysis, site of infections, use of antibacterial drugs, crude mortalities and graft losses within six months, complications after kidney transplantation, perioperative infection events and their pathogens, and antibiotic sensitivities. In addition, donor data were collected, including age, sex, cause of death, classification of utilised organs, time spent in the intensive care unit (ICU) before donation, and warm ischaemia time.

### Definitions

The classification of utilised organs was as follows: China Category I (C-I), donation after brain death (DBD); China Category II (C-II), donation after cardiac death (DCD); and China Category III (C- III), donation after brain death plus cardiac death (DBCD) [23]. DDI refers to an infection occurring when the pathogen existing in the donor’s body caused the recipient to be infected with the same pathogen after organ donation through the organ transplantation process [24]. Accurate diagnosis of DDI requires highly sensitive technologies, such as whole genome sequencing and the confirmed genetic relationships between pathogens. A possible DDI was defined as an infection occurring when the pathogen in the course of the recipient infection was identical to the microorganism cultured in the PF and had the same drug sensitivity profile [21]. The criteria used in our study to define and classify infections were proposed by the Centers for Disease Control and Prevention [25]. Delayed graft function was defined as a decrease in daily serum creatinine less than 10% from the previous day for 3 consecutive days in the first postoperative week or serum creatinine failing to decrease to 400 μmol/L in the first postoperative week [21].

### Preservation fluid and method for detecting microorganisms

The graft PF consisted of a hypertonic citrate purine solution (S400, Shanghai, China) and was used for graft perfusion during organ retrieval and for preservation during graft transportation. Prior to kidney transplantation, two samples of PF (15 ml) were collected from the bag containing the kidney, and each sample was added to a sterile blood culture bottle and fungal culture flasks for microbial culture. Bacterial species were cultured and identified using the VITEK-2 system (Biomerieux, Marcy-l’Etoile, France), and the minimum inhibitory concentration was interpreted according to the breakpoint set by the Clinical and Laboratory Standards Institute [26].

### Immunosuppressive therapy and infection prevention programs

All enrolled recipients received triple immunosuppression (tacrolimus or cyclosporin A, prednisone, and mycophenolate mofetil), and some patients were induced with anti-thymocyte immunoglobulin. The recipients were given meropenem 1 g intravenous infusion to prevent infection during the operation, and cefoperazone sulbactam sodium was given for infection prevention after the operation for at least 7 days. At the same time, antifungal prophylaxis was administered for 2 weeks, and the postoperative antimicrobial treatment was adjusted according to the microbial resistance spectrum identified in the recipient specimens and PF.

### Statistical analysis

Statistical analysis was performed using SPSS software [Version 25.0; SPSS Inc., Chicago, IL, USA]. Continuous variables with a normal distribution are described as the means and standard deviations; otherwise, they are represented as the medians and interquartile ranges (IQRs). The Kolmogorov-Smirnov test was used to assess variable distributions. Independent-sample t tests were used to compare quantitative variables with a normal distribution between groups. When quantitative variables did not follow a normal distribution, the Mann-Whitney test for nonparametric variables was used for comparisons between groups. Categorical variables are presented as frequencies and percentages. The chi-square test or Fisher’s exact test was used for the comparison of categorical variables between groups, as appropriate. For all tests, *P* < 0.05 was defined as statistically significant.

## Results

A total of 337 recipient underwent kidney transplantation between December 2018 and December 2020, including 212 recipients of organs from deceased donors and 110 corresponding deceased donors. A total of 206 PF samples were collected, and 20 recipients had CRKP-positive PF cultures. The incidence of death or graft loss in recipients with CRKP-positive PF cultures was 6/20(30.0%), and the incidence of death or graft loss in recipients with CRKP- negative PF cultures was 15/186(8.1%).

### Characteristics of deceased donors

During the study, twenty CRKP-positive recipient kidneys derived from 11 deceased donors were cultured in PF. The characteristics of the donors are shown in Table 1. There was a significant difference in the length of ICU stay between CRKP-positive kidneys cultured in PF and CRKP-negative kidneys cultured in PF (*P* < 0.05), but there were no significant differences in sex, age, cause of death, China classification of donation or warm ischaemia time. In both groups of donors, brain trauma was the most common cause of death, followed by cerebrovascular accidents. The vast majority of donors were classified as China Category III.

**Table 1:** Baseline characteristics of donors with or without a CRKP-positive preservation fluid culture

Characteristics	Donors with CRKP-positive PF	Donors without CRKP-positive PF	***P*** Value
	***N*** = 11	***N*** = 93	
Sex, male n (%)	7(63.6)	51(54.8)	0.751
Age (years)	50.82 ± 9.19	48.57 ± 9.93	0.476
**Cause of death n (%)**			
Brain trauma	6(54.5)	51(54.8)	0.985
Cerebrovascular accidents	3(27.3)	19(20.4)	0.696
Brain tumour	1(9.1)	11(11.8)	0.788
Others	1(9.1)	12(12.9)	0.718
**China classification of donation n (%)**			
I	1(9.1)	17(18.3)	0.685
II	1(9.1)	21(22.6)	0.450
III	9(81.8)	55(59.1)	0.197
ICU stay in days, median (IQR)	30(21-39)	11(9-14)	P<0.001
Warm ischaemia time (min), median (IQR)	11(10-13)	11(9-14)	0.928

### Comparison of recipients with graft loss or death and recipients without graft loss or death when PF culture was CRKP-positive

A total of 20 recipients were included in the study, with an average age of 44.3 years, 15 of whom were male. The incidence of pdd-CRKP infection was 7.3% (15/206). The most common aetiology of kidney failure was glomerulonephritis, and 15 recipients developed pdd-CRKP infection. The drug sensitivity profiles of CRKP cultured in the recipients were the same as those in the PF, and the infections occurred shortly after the operation, with an average time of 7.7 days. The difference between the two groups in the use of antibiotics, including ceftazidime-avibactam (CAZ-AVI), was statistically significant (*P* < 0.05), suggesting that the use of antibiotics, including CAZ-AVI, was a potential protective factor against graft loss or death in recipients. Due to the limited sample size, univariate and multivariate analyses could not be performed. The characteristics of the recipients are shown in Table 2.

**Table 2:** Baseline characteristics of recipients with a CRKP-positive preservation fluid culture

Characteristics	Recipients with graft loss or death	Recipients without graft loss or death	***P*** Value
	***N*** = 6	***N*** = 14	
Sex, male n (%)	5(83.3)	10(71.4)	0.573
Age (years)	36.8 ± 8.9	36.0 ± 11.8	0.879
BMI (kg/m2)	22.5 ± 3.8	22.4 ± 3.6	0.948
**Aetiology of kidney failure, n (%)**			
HTA	1(16.7)	1(7.1)	0.521
DM	1(16.7)	1(7.1)	0.521
Glomerulonephritis	4(66.7)	11(78.6)	0.613
Others	0(0.0)	1(7.1)	0.502
**Type of dialysis n (%)**			
HD	4(66.7)	12(85.7)	0.549
PD	2(33.3)	2(14.3)	0.549
**Duration of dialysis (years) median (IQR)**			
Duration of HD	1.5(0.0-5.5)	2.0(0.4-5.0)	0.588
Duration of PD	0.0(0.0-1.8)	0.0(0.0-0.0)	0.345
DGF n (%)	1(16.7)	5(35.7)	0.613
ATG induction n (%)	2(33.3)	5(35.7)	0.919
Pdd-CRKP infection (%)	6(100.0)	9(64.3)	0.091
Antibiotic regimen containing ceftazidime avibatan (%)	1(16.7)	11(78.6)	0.018

*Abbreviations*: *BMI* body mass index, *HTA* hypertension, *DM* diabetes mellitus, *HD* haemodialysis, *PD* peritoneal dialysis, *DGF* delayed graft function, *ATG* anti-thymocyte globulin

### Impacts of pdd-CRKP infection on recipients and outcomes

The kidneys of 10 recipients with pdd-CRKP infection were derived from six donors, and six liver transplantation recipients from these six donors also had isolated CRKP with the same drug susceptibility profile. The other 5 recipient kidneys with pdd-CRKP infection were derived from three other deceased donors. Two of the donors did not perform liver donation, and the other donors’ liver recipients did not cultivate CRKP. The isolation of the same drug-sensitivity profile of CRKP from six liver recipients provided stronger evidence of CRKP transmission from donors to recipients. However, none of these CRKP-infected recipients could be classified as having a proven DDI, and all 15 CRKP-infected recipients were classified as having a possible DDI. The infection characteristics of the patients with pdd-CRKP infection are shown in Table 3. The most common type of infection was surgical site infection, followed by bloodstream infection. Among the 15 recipients, 3 underwent graft nephrectomy due to CRKP infection. In addition, three recipients died, one due to rupture of the graft artery and two due to septic shock.

**Table 3:** Frequency and incidence of infection in recipients with a CRKP-positive preservation fluid culture

Infectious events	Number of cases (n)	Incidence (*n* = 20)
Surgical site infection	11	55.0%
Infectious allograft kidney artery disruption	1	5.0%
Urinary tract infection	3	15.0%
pneumonia	2	10.0%
Bloodstream infection	10	50.0%

### Antibiotics protocol for CRKP-positive culture in PF

At the time of transplantation, PF culture results had not yet been reported, so the standard antimicrobial drug prevention regimen was adopted in our centre. When the PF culture results were reported, the antimicrobial drug regimen was adjusted according to the drug sensitivity results. The antibiotics selected in the 20 recipients with positive CRKP in recipient PF cultures are shown in Table 4. Eight of the 20 recipients received tigecycline combined with meropenem or imipenem for prophylactic anti-infection, 4 received CAZ-AVI alone, 5 received CAZ-AVI combined with meropenem, and 3 were first given tigecycline combined with imipenem and then CAZ-AVI for salvage treatment. Three deaths and two graft losses occurred during the prophylactic anti-infective regimen of tigecycline combined with meropenem or imipenem. The other surviving recipients underwent graft nephrectomy with tigecycline combined with imipenem after failing to respond to anti-infective therapy, followed by salvage therapy with CAZ-AVI. Two of the four recipients of ceftazidime alone on a prophylactic anti-infective regimen developed surgical incision infection but recovered successfully. Among the 5 recipients who used CAZ-AVI combined with meropenem as a preventive anti-infective regimen, although 2 cases of incision infection and one case of bacteremia occurred, they all recovered smoothly after anti-infective treatment. The spectrum of susceptibility of CRKP strains isolated from recipients with pdd-CRKP infection is shown in Table 5.

**Table 4:** Prophylactic antimicrobial therapy regimens in recipients with a CRKP-positive preservation fluid culture

Prophylactic antimicrobial therapy regimens(n)	Death(n)	Graft loss(n)	Patient and graft survival(n)
Tigecycline+ imipenem / meropenem (8/20)	3	2	3
CAZ-AVI + meropenem (5/20)	0	0	5
CAZ-AVI as initial therapy (4/20)	0	0	4
CAZ-AVI as salvage therapy (3/20)	0	1	2

*Abbreviations*: *CAZ-AVI* Ceftazidime-Avibatan

**Table 5:** Antimicrobial susceptibility of isolates from recipients with pdd-CRKP infections

Antibiotic	Susceptible (%)
Ceftazidime	0.0
Levofloxacin	13.3
Gentamycin	20.0
Imipenem	0.0
Meropenem	0.0
Amikacin	40.0
Polymyxin	86.7
Tigecycline	93.3
Ceftazidime-Avibatam	100.0

## Discussion

Although numerous reports have shown that approximately 3% of SOT recipients are affected by fatal DDI, the use of organs from marginal donors is increasing due to the current organ shortage [11, 27]. The incidence of DDI is reduced by donor screening and the use of prophylactic antimicrobials in recipients, but transmission can still occur and may lead to increased morbidity and mortality [28]. The PF is a possible pathway for DDI. Most of the microorganisms in PF are coagulase-negative *Staphylococci* and *Enterococci* [20, 21], and most of them are contaminated saprophytic flora. Because of the widespread use of prophylactic antibiotics in transplant recipients, these low-virulence organisms are difficult to transmit through PF, resulting in significantly reduced pathogenicity [29]. However, MDR *Enterobacteriaceae* are more virulent and more likely to transmit through PF [30, 31]. Nevertheless, there are few data on the donor-derived transmission of MDR gram-negative bacilli through PF, especially CRKP. To our knowledge, this report represents the largest study of kidney transplant recipient DDIs caused by CRKP to date.

In our study, a 7.3% (15/206) incidence of pdd-CRKP infection was associated with PF, and the risk of total infection events due to PF contamination ranged from 0.83 to 14.3% in previous reports [32, 33]. However, only CRKP in PF was investigated, and DDIs caused by other microorganisms in PF were not investigated in this study. The rate of positive CRKP culture in PF in our centre is high, which may be related to the high incidence of CRKP in our centre. In China, CHINET data showed that the resistance rates of *Klebsiella pneumoniae* to imipenem and meropenem rose steadily from 9.2 and 9.2% in 2010 to 23.3 and 24.2% in 2020, respectively. The detection rate of CRKP in Anhui Province, where our centre is located, increased steadily from 8.9% in 2010 to 25% in 2020. On the other hand, in our cohort, the ICU stay of donors in the CRKP-positive PF culture group was longer than that in the CRKP-negative PF culture group, and the difference was statistically significant. Prolonged (> 7 days) ICU stays, the use of vasopressors, and the need for cardiopulmonary resuscitation were reported to be independent risk factors for predicting potential infections in donors [34]. Studies have found that a hospital stay of as little as 2 days is sufficient to acquire MDR hospital pathogens that can be transmitted by transplantation [35]. In our study, the age of donors with CRKP-positive PF cultures was generally higher, with an average age of 50.8 years. A prospective study by Oriol et al. found that higher donor age was an independent risk factor for positive culture of high-risk microorganisms in PF [36]. Microbial contamination of the ICU environment and intestinal damage during the organ retrieval process may also contaminate the PF and lead to a higher positive rate of CRKP cultures.

Whether microbial contamination of PF in SOT is related to DDI is controversial. Bertrand et al. reported a negligible incidence of clinical complications due to PF contamination [20]. However, some other studies have suggested that it is a common cause of early posttransplant infection [29, 37]. The occurrence of DDI also depends on the type of contaminating microorganism. MDR *Klebsiella pneumoniae*, *MDR Acinetobacter baumannii* and other bacteria are more likely to cause DDIs associated with PF than other sensitive *Cocci* species [21]. Cai et al. reviewed the literature on donor-derived CRKP infection from 2011 to 2018, including a total of 18 donor-derived CRKP infection recipients. Postoperatively, 1 patient died early (9 days), 5 patients died late (≥28 days), and 1 patient lost the graft kidney due to rupture of the graft artery. The overall mortality was 33.3% [13], which was higher than the overall mortality of pdd-CRKP infection in our centre (20%).

Most of the DDIs associated with PF were surgical site infections, including local infection of the graft site, infectious renal graft artery rupture and wound infection [21], which was consistent with the results of our centre. The most common type of infection in our centre was surgical site infection, and 11 of the 20 recipients had surgical site infection, followed by bloodstream infection. CRKP bloodstream infections have been reported to be the most fatal type of infection, with a mortality of up to 20-50% [38, 39]. A total of 10 CRKP-positive recipients developed bloodstream infection in our centre. Among these 10 CRKP-positive bloodstream infection recipients, there were 2 deaths and 3 graft losses. One of the recipients died of graft artery rupture, a fatal complication of donor-derived CRKP infection, which has been reported in both liver and kidney recipients with donor-derived CRKP infection [13, 40].

Due to the lack of evidence, there is no broad consensus regarding the treatment recipients with of CRKP-positive cultures in PF. Some authors have suggested that when MDR is detected in donors and PF, targeted antibacterial treatment should be performed after transplantation, which could prevent the transmission of MDR gram-negative bacteria. DDIs have been reported despite antibiotic prophylaxis [41, 42]. The authors also suggested that extended antibiotic use for 10 days after surgery might help reduce the risk of transmission and associated mortality [19]. Our study confirmed the clinical efficacy of CAZ-AVI in the prevention and treatment of CRKP infection. None of the 9 recipients who were initially given prophylactic anti-infective therapy with CAZ-AVI alone or CAZ-AVI combined with meropenem died or had graft loss. It has been reported that CAZ-AVI treatment is the only independent predictor of survival in CRKP-infected patients [43], and recent clinical trials have also demonstrated that CAZ-AVI-based therapy has successfully treated CRKP infection in immunocompromised populations [44]. In addition to drug control of infection, effective source control, such as allogeneic nephrectomy and complete debridement, can increase the success rate of cured infections [45–47]. Allograft nephrectomy not only provides complete debridement but also reduces the bacterial load. In our study, all three surviving recipients with severe CRKP infection received allograft nephrectomy, complete debridement and drainage, and all achieved good outcomes.

Test protocols for the rapid detection of pathogens carried by donors await further study. The time required for routine microbial culture in PF from specimen collection to final results (including drug susceptibility results) may range from 48 h to 120 h, depending on the testing time and the need for additional resistance testing. This delay affects the time to start appropriate antimicrobial therapy. Although the polymerase chain reaction method can quickly obtain test results, it is expensive, has limited specificity and cannot provide information about the sensitivity of the drug. Delay in communication is another important factor in the transmission of infection [48]. Effective and timely communication between the organ procurement organisation (OPO) and the transplant centre is essential to control the transmission of pathogens and should be conducted within 24 h. Therefore, routine sample culture (blood, urine, and tracheal secretions) of potential donors in hospitals in areas where CRKP pathogens are endemic is recommended for the rapid identification of pathogens [9]. Rapid communication between the organ transplant centre and the OPO is also necessary, and it can play an important role in accelerating the use of targeted antibiotic treatment in patients who receive organs from infected donors.

The study has several limitations. First, the lack of routine microbiological screening of donors made it difficult to determine the source of CRKP and the relationship between donor infection and donor-derived CRKP infection after transplantation. Second, there were no data on the use of antibiotics given to donors. Third, regarding the transmission of DDI, genotyping technology provides necessary objective evidence for this event. Unfortunately, this technology is not widely used in our hospital. Therefore, our detection algorithm may overestimate transmission events. Finally, our sample size was small, mainly because of the low incidence of donor-derived CRKP infection, and our results may not be applicable to other transplant centres.

## Conclusion

In summary, this study showed that the incidence of pdd-CRKP is high in our centre. Recipients with pdd-CRKP infection can still achieve a good prognosis with the use of antimicrobial agents including CAZ-AVI and donor screening for CRKP should be included in measures to contain the incidence of DDI.

## Supplementary Information

**Additional file 1.**

**Additional file 2.**

## Data Availability

All data generated or analysed during this study are included in this published article [and its supplementary information files].

## Abbreviations

CRKP: Carbapenem-resistant *Klebsiella pneumoniae*
DDI: Donor-derived infections
PF: Preservation fluid
ICU: Intensive care unit
KT: Kidney transplant
MDR: Multidrug resistance
pdd-CRKP: possible donor-derived carbapenem-resistant *Klebsiella pneumoniae*
SOT: Solid organ transplantation
